# Can we predict hypoadrenocorticism in dogs with resting hypocortisolemia? A predictive model based on clinical, haematological, and biochemical variables

**DOI:** 10.3389/fvets.2024.1523170

**Published:** 2024-12-23

**Authors:** Nuno Sousa Santos, Tiago Dias Domingues, Antonio Maria Tardo, Marta Dinis, Luísa Mateus, Federico Fracassi, Rodolfo Oliveira Leal

**Affiliations:** ^1^Faculty of Veterinary Medicine, University of Lisbon, Lisbon, Portugal; ^2^CEAUL, Faculdade de Ciências, University of Lisbon, Lisbon, Portugal; ^3^Department of Veterinary Medical Sciences, University of Bologna, Bologna, Italy; ^4^Faculty of Veterinary Medicine, Veterinary Teaching Hospital, University of Lisbon, Lisbon, Portugal; ^5^Associate Laboratory for Animal and Veterinary Sciences (AL4AnimalS), Faculty of Veterinary Medicine, CIISA – Centre for Interdisciplinary Research in Animal Health, University of Lisbon, Lisbon, Portugal

**Keywords:** ACTH stimulation test, Addison’s disease, chronic enteropathy, hypoadrenocorticism, resting cortisol

## Abstract

**Background:**

A resting cortisol concentration (RC) higher than 2.0 μg/dL (55 nmol/L) is commonly used to rule out hypoadrenocorticism (HA). However, there is a significant overlap of RC between dogs with HA and those with other diseases. There is a need for data to help increase the suspicion of HA.

**Hypothesis/objectives:**

To create a predictive model based on clinical, haematological and biochemical variables to increase the likelihood of diagnosis of hypoadrenocorticism.

**Animals:**

Ninety-two dogs with RC <2.0 μg/dL, and an adrenocorticotropic hormone stimulation test (ACTHst) performed.

**Methods:**

Multicentric retrospective cohort study with review of medical records of client-owned dogs presented to two referral centres, between January 2018 and March 2022. Dogs were divided into two groups (HA and not HA), based on ACTHst results. Descriptive analysis was performed along with a predictive model, using univariable and multivariable logistic regression analysis.

**Results:**

Of the 92 included dogs, hypoadrenocorticism was diagnosed in 29 patients (32.2%) based on ACTHst results (HA group). Acute gastrointestinal signs, anorexia and lethargy were more prevalent in the HA group. Creatinine, BUN, ALT, and potassium were higher, and albumin, sodium and Na/K ratio were lower in the HA group. Multivariate analysis developed a robust model in which lethargy (OR 5.25), anorexia (OR 3.69), albumin (OR 0.32), and sodium (OR 0.84) concentrations allowed the prediction of HA.

**Conclusions and clinical importance:**

In dogs with resting hypocortisolemia, the combination of anorexia and lethargy, along with low sodium and albumin concentrations, should raise the suspicion of hypoadrenocorticism. The variables identified in this study may help clinicians to identify dogs with hypoadrenocorticism in daily clinical practice.

## Introduction

The estimated prevalence of naturally occurring hypoadrenocorticism (HA) in dogs is between 0.06 and 0.28% (1, 2), and the clinical signs are often vague including lethargy, vomiting, anorexia, diarrhoea, weakness, and weight loss (2, 3). As a result of its non-specific clinical presentation, there is a significant overlap of clinical signs with other conditions, namely chronic gastrointestinal diseases. Measurement of resting cortisol concentration (RC) is commonly performed as a screening test for HA (4, 5). Due to its high sensitivity, a RC is often performed and a cortisol higher than 2.0 μg/dL (55 nmol/L) allows clinicians to rule out HA (4, 5). Although a recent study (6) documented a prevalence of eunatremic, eukalemic hypoadrenocorticism (EEH) as low as 0.9% in dogs with chronic gastrointestinal signs, RC is still routinely performed in patients with chronic gastrointestinal disease.

Two other recent studies assessed the prevalence of HA in dogs with chronic gastrointestinal signs, at referral hospitals (7, 8). Taking both studies, out of the 433 dogs, 121 (28%) had RC concentrations <2.0 μg/dL (<55 mmol/L), with 34 (8%) having concentrations RC <1.0 μg/dL (<27.5 mmol/L). Despite these percentages, only 7 dogs (1 dog in one of the studies, and 6 in the other), had a diagnosis of HA, corresponding to a combined prevalence of 1% (7, 8). Tardo et al. (6) focused on the overlap of cases with EEH and other conditions, presenting with chronic gastrointestinal signs, highlighting increased suspicion of EEH in dogs with higher endogenous ACTH concentration and lower cortisol-to-ACTH ratio (6). These studies supported that there is a significant overlap of RC between dogs with HA and other diseases, stressing the need for data that will lead to an increased suspicion of HA, alongside the measurement of RC in order to reduce unnecessary testing.

This study aimed to retrospectively characterise dogs with RC < 2.0 μg/dL (<55 nmol/L), assessing the value of clinical and laboratorial findings as predictors of post-ACTH cortisol result.

## Materials and methods

### Study design and data

A multicentric retrospective cohort study was conducted. Dogs consulted in two veterinary teaching hospitals of southern Europe (Veterinary Teaching Hospital of the University of Bologna and Veterinary Teaching Hospital of the University of Lisbon) from January 2018 to March 2022 were recruited.

Digital databases of medical records were retrospectively assessed to identify dogs submitted to RC measurement. From these, all cases with RC <2.0 μg/dL (<55 mmol/L) and available ACTH stimulation test (ACTHst) results were included. Cases with previous diagnosis of HA, those which had received glucocorticoids in the previous 8 weeks and dogs with a diagnosis of hypercortisolism were excluded.

Data collected from each patient included age, gender, breed, weight, clinical signs, haematology results, biochemistry results including electrolytes, ultrasound measurement of adrenal glands, urine specific gravity, resting cortisol concentration, post-ACTH cortisol concentration and, when available, endogenous ACTH concentration. According to the ACTH stimulation test results, cases were divided into two groups: (1) dogs diagnosed with HA (if post-ACTH cortisol concentration was <2.0 μg/dL) and (2) dogs in which HA was excluded (nHA group).

Resting and post-ACTH cortisol concentrations were assessed by chemiluminescence (Immulite 1,000 or 2000, Siemens) at the endocrinology laboratory of each institution, both approved by the standards of Quality of the European Society of Veterinary Endocrinology.

Blood samples for cortisol measurement were collected in plain tubes, centrifuged for 10 min at 3000 g and either stored at 4°C for same day analysis or at −20°C until sample processing (usually in the next 48–72 h). For the ACTH stimulation test, a blood sample was collected before intravenous or intramuscular injection of synthetic ACTH (Synachten, Alfasigma or Cosacten, Dechra Laboratories) at a dose of 5 μg/kg, and another sample was collected 1 h after the injection.

### Statistical analysis

Quantitative variables were described as median and range (min-max values). The underlying normality of the data was assessed using a Kolmogorov–Smirnov test with Lilliefors correction. Statistical comparisons between two independent groups were performed using Mann–Whitney U test for continuous variables. Regarding qualitative variables, these were described as frequencies and percentages. Chi-square test or Fisher exact test was used to assess associations between qualitative variables.

To evaluate the impact of clinical and laboratory variables in hypoadrenocorticism, multivariable binary logistic regression was constructed considering the HA (0—without disease, 1—with disease) as the dependent variable. Stepwise backwards selection using the Akaike Information Criterion (AIC) was used to select the best set of variables that explain the response variable. Multicollinearity was assessed through the Variance Inflation Factor (VIF) considering a cut-off value of 5 (9). Odds ratio (OR) and respective confidence intervals were also presented. To evaluate the performance of the model we also computed the receiver operating characteristic (ROC) curve to differentiate between disease cases and non-disease cases. We then estimated the respective areas under these curves (AUC) and computed their 95% confidence intervals. The Hosmer–Lemeshow statistic was also presented.

Univariable and multivariable binary logistic regression models were performed in order to predict HA. The variables that in the univariable analysis yielded a *p*-value <0.15 were included in the multivariable logistic regression model (10).

Statistical analysis was performed using the software R version 4.2.2. All the results with a *p <* 0.05 were considered statistically significant.

## Results

A total of 90 dogs were included, of which 47 were females (52.2%). The median age was 4.0 years (0.25–14). The median weight was 19.8 kg (1.6–48). According to ACTH stimulation test results, 29/90 (32.2%) cases were diagnosed with HA (HA group).

In the HA group (n = 29), there were 18 (62%) females of which 15 (83%) were neutered. From the 11 (38%) males, 6 were neutered (55%). There were 13 mixed breed dogs, 2 Border Collies, 2 Jack Russel Terriers and 1 each of 12 other breeds.

In the nHA group (n = 61), there were 29 (47.5%) females of which 17 were neutered (57%). From the 32 males (52.5%), 20 were entire (63%). There were 15 mixed breed dogs, 5 German Shepherd, 4 Golden Retrievers, 4 Portuguese Water Dog, 4 Labrador Retrievers, 3 Boxers, 3 Jack Russel Terrier, 2 Weimaraner, 2 Yorkshire Terrier and 1 each of 19 other breeds.

No differences were observed between sex and diagnosis of HA (chi-squared test: *χ*^2^ = 1.13, df = 1, *p* = 0.29) (Table 1). The median age was 5 (0.8–10.75) years in the HA group and 3.0 (0.25–14) in the nHA group (*p* = 0.05). Median weight was 15 (1.6–48) kg and 21.8 (2.75–46.4) kg in the nHA group and the HA group, respectively (*p* = 0.07) (Table 1).

**Table 1:** Sample characteristics considering total sample and disease status.

Variable	Total (*n* = 90)	HA group (*n* = 29)	nHA group (*n* = 61)	*p*-value
Sex (*n*, %)				
Female	47 (52.2)	18 (62.1)	29 (93.5)	0.29*
Male	43 (47.8)	11 (37.9)	32 (52.5)	
Age (years) Median (IQR)	4.0 (5.75)	5.0 (3.0)	3.0 (7.0)	0.05**
Body weight (kg) Median (IQR)	19.82 (18.82)	15 (14.5)	21.8 (17.4)	0.07**

*Chi-square test; **Mann–Whitney *U* test.IQR, interquartile. *p*-values were obtained by comparisons between HA group vs. nHA group.

### Clinical signs

In the HA group, 17 dogs (59%) presented with acute gastrointestinal signs (e.g., diarrhoea, vomit, regurgitation) while 12 (41%) showed a chronic history of gastrointestinal signs. Six dogs (21%) were diagnosed on the first gastrointestinal episode. In the nHA group, there were 9 cases (15%) of acute and 43 cases (70%) of chronic gastrointestinal signs, 3 dogs (5%) in this group were tested on the first gastrointestinal episode. Regarding non gastrointestinal clinical signs, in the HA group, polyuria and polydipsia was identified in 8 dogs (27.5%), lethargy in 22 (76%) and anorexia in 17 (59%), compared to the nHA group with 6 (10%), 12 (20%) and 12 (20%), respectively (Table 2). Shock signs were grouped together for the assessment and were considered present when there was a combination of tachycardia, weak pulses or hypotension, hypothermia or altered mentation.

**Table 2:** Clinical signs by disease status.

Variable	Total (*n* = 90)	nHA group (*n* = 61)	HA group (*n* = 29)	*P*-value
Acute gastrointestinal signs (*n*, %)	26 (29%)	9 (15%)	17 (59%)	<0.001*
Chronic Gastrointestinal signs (*n*, %)	55 (61%)	43 (70%)	12 (41%)	0.016*
Neurological signs (*n*, %)	8 (9%)	6 (10%)	2 (7%)	0.95*
Polyuria/Polydipsia (*n*, %)	14 (16%)	6 (10%)	8 (27.5%)	0.06*
Lethargy (*n*, %)	34 (38%)	12 (20%)	22 (76%)	<0.001*
Anorexia (*n*, %)	29 (32%)	12 (20%)	17 (59%)	<0.001*
First gastrointestinal episode (*n*, %)	9 (10%)	3 (5%)	6 (21%)	0.03**
Shock signs (*n*, %)	10 (11%)	0 (0.0%)	10 (35%)	<0.001**

*Chi-square test; **Fisher exact test. *p*-value were obtained by comparisons between HA group vs. nHA group.

### Haematology, serum biochemistry, and urinalysis

In this study, median lymphocyte count was higher in the HA group, with a median of 2,840/uL (727–7,070), compared to 2,048/uL (848–8,507) in the nHA group, but the difference between groups was not statistically significant (*p* = 0.05). Other haematological parameters were similar between groups. Data regarding full haematology was not available for one of the dogs in HA group and two in the nHA group.

**Figure 1:**
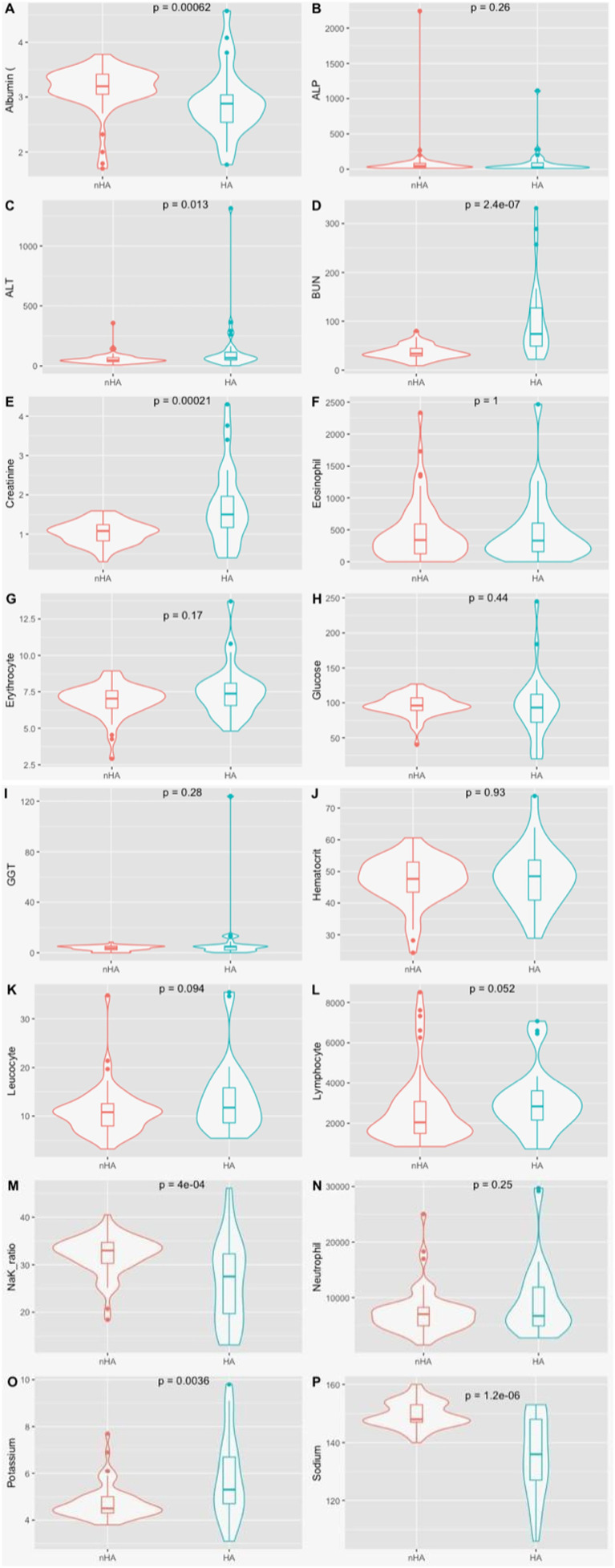
Violin plot for the clinical variables by disease status. **(A)** Albumin (g/dl); **(B)** ALP, alkalin phosphatase (U/L); **(C)** ALT, alanine aminotransferase (U/L); **(D)** BUN, blood urea neutrogen (mg/dl); **(E)** Creatinine (mg/dl); **(F)** Eosinophil Count (absolute values); **(G)** Erythrocyte count (M/μL); **(H)** Glucose (mg/dl); **(I)** GGT, gamaglumatiltransferase (U/L); **(J)** Haematocrit (%); **(K)** Leukocyte count (x10^3^/ μL); **(L)** Lymphocyte count (absolute value); (M) Na/K Ratio; **(N)** neutrophils count (absolute value); **(O)** Potassium (mmol/L); **(P)** Sodium (mmol/L); P denotes the *p*-value of the Mann whitney *U* test.

Serum biochemistry results showed significant differences in albumin, with lower median levels in the HA group, in comparison with the nHA (2.88 vs. 3.2, *p* < 0.001). Median levels of BUN (74 vs. 33.8, *p* < 0.001), ALT (67 vs. 46.5, *p* = 0.001) and creatinine (1.50 vs. 1.08, *p* < 0.001), were significantly higher in the HA group (Figure 1; more detailed results can be found in Supplementary Table A1). In the HA group, one patient did not have full biochemistry results available, 7 others did not have GGT results available, another did not have ALP results. In the nHA group, there wasn’t available data regarding GGT in 17 cases, ALT and ALP in 2 cases, creatinine in 1 case, BUN in 2 cases, glucose in 3 and albumin in 2 cases.

In the HA group, 21/29 (72.4%) were cases of hyponatremic and/ or hyperkalemic HA. Hyperkalemia was considered with values of potassium over 5.8 mmol/L, hyponatremia was considered with values of sodium under 144 mmol/L. The remaining cases, (8/29) were diagnosed with EEH.

In the nHA group, 5 dogs were hyperkalemic, 6 were hyponatremic, with 2 being both hyperkalemic and hyponatremic. Sodium/ potassium ratio was calculated for every patient. In the nHA group the median was 33, ranging from 18.4 to 40.5, while in the HA group, the median was 27.5, ranging from 13.1 to 46.1. Sodium and potassium levels and the ratio were significantly different between groups, with *p* < 0.001, *p* = 0.004 and *p* < 0.001, respectively. One patient in this group did not have available data regarding electrolytes.

Data regarding urine specific gravity was available in 30 dogs of the nHA group and in 17 dogs of the HA group. Values ranged from 1,005 to 1,072, with a median of 1,035, in the nHA group. On the HA group, values ranged from 1,010 to 1,070, with a median of 1,024. There was no statistically significant difference between both groups (*p* = 0.25).

### Resting serum cortisol concentrations and endogenous ACTH

Among the 90 dogs showing RC < 2.0 μg/dL, 36 (40%) had a value <1.0 μg/dL while 54 (60%) showed a RC between 1.0 and 2.0 μg/dL. There was a significant difference between the percentage of dogs in each group with RC < 1.0 μg/dL, detailing 10 (16%) of dogs in the nHA group and 26 (90%) in the HA group (Chi-square test: *p* < 0.001).

Endogenous ACTH results were available for 13 dogs in the nHA group and for 13 dogs in the HA group. In the first group, the median was 20 pg./mL, ranging from 5 pg./mL to 25.3 pg./mL. In the HA group, the median was 487 pg./mL, ranging from ranging from 5 pg./mL to 1,250 pg./mL. The value of endogenous ACTH was significantly higher in the HA group (*p* < 0.002).

### Predictors of hypoadrenocorticism

Univariable and multivariable logistic regression results are shown in Table 3. We observed that the variables lethargy, anorexia, albumin, and sodium were predictors of HA (Table 3). The variables ALT, BUN, creatinine, leucocytes, and neutrophils were excluded from the multivariable logistic regression model since they show VIF > 5.

**Table 3:** Univariable and multivariable logistic regression results considering hypoadrenocorticism as the dependent variable.

Univariable logistic regression model					Multivariable logistic regression model			
Variable	*β*	SE	OR (CI 95%)	*p*	*β*	SE	OR (CI 95%)	*p*
Male vs. Female	0.59	0.46	1.81 (0.74–4.56)	0.19	–	–	–	–
Age (years)	0.08	0.07	1.08 (0.95–1.23)	0.24	–	–	–	–
Weight (kg)	−0.03	0.02	0.97 (0.92–1.01)	0.11	–	–	–	–
Entire vs. Neutered	1.06	0.49	2.89 (1.14–7.89)	0.03	–	–	–	–
Acute Gastrointestinal Signs (yes)	2.10	0.52	8.19 (3.03–23.78)	<0.001	–	–	–	–
Chronic gastrointestinal signs (yes)	−1.22	0.47	0.29 (0.11–0.73)	0.009	–	–	–	–
Neurological signs (yes)	−0.39	0.85	0.68 (0.09–3.18)	0.65	–	–	–	–
Polyuria/Polydipsia (yes)	1.25	0.59	3.49 (1.09–11.79)	0.04	–	–	–	–
Lethargy (yes)	2.55	0.54	12.83 (4.66–39.46)	<0.001	1.66	0.73	5.25 (1.26–23.36)	0.02
Anorexia (yes)	1.76	0.49	5.78 (2.23–15.78)	<0.001	1.31	0.79	3.69 (0.78–18.75)	0.09
First gastrointestinal episodes (yes)	1.62	0.75	5.04 (1.22–25.52)	0.03	–	–	–	–
Albumin	−1.13	0.49	0.32 (0.11–0.83)	0.02	−1.15	0.67	0.32 (0.08–1.16)	0.09
ALP	−3.79e-5	8.84e-4	0.99 (0.99–1.00)	0.97	–	–	–	–
ALT	0.008	0.004	1.01 (1.00–1.02)	0.04	–	–	–	–
BUN	0.06	0.02	1.07 (1.04–1.11)	<0.001	–	–	–	–
Creatinine	2.24	0.64	9.37 (3.13–39.03)	<0.001	–	–	–	–
Eosinophil count	7.03e-5	4.75e-4	1.00 (0.99–1.00)	0.88	–	–	–	–
Erythrocyte count	0.35	0.19	1.42 (1.01–2.14)	0.06	–	–	–	–
Glucose	−0.003	0.008	0.99 (0.98–1.01)	0.71	–	–	–	–
GGT	0.16	0.09	1.17 (1.01–1.42)	0.08	–	–	–	–
Haematocrit	0.011	0.03	1.01 (0.96–1.07)	0.68	–	–	–	–
Leucocyte count	0.08	0.04	1.08 (1.00–1.18)	0.06	–	–	–	–
Lymphocyte count	0.0002	0.0001	1.00 (0.99–1.00)	0.25	–	–	–	–
Sodium/potassium ratio	−0.15	0.04	0.86 (0.79–0.93)	<0.001	–	–	–	–
Neutrophil count	8.58e-5	4.66e-5	1.00 (0.99–1.00)	0.07	–	–	–	–
Potassium	0.74	0.23	2.09 (1.39–3.42)	0.001	–	–	–	–
Sodium	−0.21	0.05	0.81 (0.73–0.88)	<0.001	−0.18	0.05	0.84 (0.74–0.91)	<0.001

β - regression coefficient; SE, standard error; OR, odds ratio: CI 95%, confidence interval at 95%. For clinical signs the reference category considered was 0 (negative response).

Dogs with lethargy and anorexia have 5.2 and 3.2 times more chance to have HA, respectively (Table 3). In addition, lower values of albumin and sodium were risk factors for HA, respectively [OR = 0.32 (0.08–1.16); OR = 0.84 (0.74–0.91)] (Table 3). The model discriminates well the disease cases (AUC = 0.92 (CI 95%: 0.85–0.99); Hosmer-Lemeshow statistic: 5.47, *p* = 0.71).

## Discussion

This study explored clinical and laboratorial parameters in dogs with low RC (< 2.0 μg/dL), to help clinicians prioritise the medical investigation of HA. We observed that lethargy, anorexia, albumin, and sodium may support an increase in the suspicion of this endocrine disease.

In our cohort of dogs, HA was confirmed in only 32% of cases. This result reinforces that resting hypocortisolemia is more often associated with a low-peak cortisol rather than hypofunctional adrenal glands. These results are in agreement with recent studies showing that HA has a low prevalence, even in dogs with RC <2.0 μg/dL (3).

In the present study, in line with the pathophysiology and presentation of the disease, most dogs diagnosed with HA showed acute gastrointestinal signs, lethargy and anorexia. In the HA group there were also 10 cases with reported shock signs, in contrast with the nHA group, in which none of the patients had this presentation. Although chronic gastrointestinal signs can occur in canine HA, chronic signs were more frequent in the nHA group. This agrees with previous literature documenting a low prevalence of HA in dogs with chronic gastrointestinal signs, even though the routine measurement of RC in those cases is still conducted (6–8). Out of the 12 dogs in the HA group with chronic gastrointestinal signs, only 4 showed hyperkalemia and hyponatremia. The remaining cases did not present electrolytic abnormalities, showing normal sodium/potassium ratios. This suggests that chronic gastrointestinal signs seem to be more often associated with EEH, despite its rare prevalence (6). It is also worth to note that the presence of chronic gastrointestinal signs reduced the likelihood of hypoadrenocorticism, with an OR of 0.29, questioning the real need of assessing RC in patients with this clinical presentation.

Regarding RC in both groups, 90% of cases of HA presented a RC <1.0 μg/dL, while there was a higher percentage of cases with values ≥1.0 and < 2.0 in the nHA group (84%). These results showed an agreement with previous studies suggesting the use of lower values of RC as a cut-off to increase the specificity for the diagnosis of HA. It is worthwhile to mention that Gold et al. (5) suggests the use of 0.8 μg/dL as the ideal cut-point, with a sensitivity of 96.9% and a specificity of 95.7%, however there are possible limitations regarding the available analysers, not allowing for precise values under 1 μg/dL Consequently, the general cut-off value of 2.0 μg/dL to rule out the disease, although adequate and needed to clearly rule out HA, it may be too high to justify further investigations in cases with low level of suspicion.

In our study, there was no significant difference in haematological parameters between groups. Described haematology findings in patients with HA include anaemia (non-regenerative, normocytic, normochromic), neutrophilia, eosinophilia and lymphocytosis (11–13). However, only 10 to 30% of cases present these changes in previous studies (14). Our results reinforce that although haematology findings may be helpful in prioritising HA on the differentials list, they are not consistent in daily practice.

Concerning serum biochemistry, there was a significant difference between groups on the values of albumin (lower in the HA group), BUN, ALT and creatinine (higher in the HA group). These differences are in line with previous studies (15–18). The increase in creatinine and BUN, is believed to be associated with hypovolaemia, but the more significant increase in BUN, in comparison with creatinine, alongside the gastrointestinal signs, also opens the possibility of concomitant gastrointestinal bleeding. However, the logistic regression shows that increased creatinine levels are a more significant risk factor for hypoadrenocorticism (OR 9.37) compared with BUN levels (OR 1.07). The lower value in albumin is likely associated with impaired synthesis and gastrointestinal loss of protein, although its pathophysiology is not clear (3). In humans, glucocorticoids seem to be associated with the enhancement of the barrier function of epithelial cells, but it is not known if the same happens in dogs (19). High levels of albumin also showed a significant decrease in the likelihood of a diagnosis of hypoadrenocorticism with an OR of 0.32. Although not routinely measured and not available for every dog of our population, there was an expected significant difference of endogenous ACTH levels, between both groups. The significantly higher levels are particularly relevant in the context of a low basal cortisol, supporting the diagnosis of HA.

Focusing on electrolytes, there was significantly higher potassium, and lower sodium and Na/K ratio in the HA compared with nHA dogs. Hyperkalemia and hyponatremia are the most common and recognizable biochemical abnormalities in dogs with HA, and a low Na/K ratio is associated with an increase in diagnostic specificity for HA (13, 20). Cases with electrolyte changes are more frequent than those without it, reinforcing the value of sodium, potassium and Na/K in the assessment of likelihood of the disease in practice as well as its inclusion in the current model. As expected, the logistic regression analysis confirmed a high level of potassium and a low level of sodium as relevant for the diagnosis of hypoadrenocorticism, with an odds-ratio of 2.09 and 0.81, respectively. The USG was not available in all dogs, but the comparison of those available showed a lower median USG value in the HA. This difference, however, was not statistically significant.

The multivariate logistic regression model conducted in this study identified a significantly increased likelihood of a HA diagnosis in the presence of lethargy, anorexia and low levels of sodium and albumin, with that possibility increasing with lower values for these parameters. The trend of clinicopathological abnormalities identified with this analysis, allows us to increase our suspicion of hypoadrenocorticism in these cases. Consequently, it is possible to select better candidates for further adrenal function testing due to the increased probability of diagnosing hypoadrenocorticism when the patient presents with the abnormalities considered most relevant in the model. This information is particularly relevant to owners facing financial concerns. This analysis also removes possible confounding factors added by other parameters, reinforcing clinical suspicion with an assessment based on clinical history and easily accessible blood results. Cases in which the patient presents resting hypocortisolemia with normal values of albumin, and sodium, as well as no evidence of lethargy and normal appetite, can be considered unlikely to have hypoadrenocorticism, and so diagnostic approach should prioritise other differentials. Despite this model, authors still defend that ACTHst should be performed when facing a resting hypocortisolemia. However, the urgency of the test can be judged and adapted according to the estimated obtained likelihood.

This study has some limitations, detailing its retrospective nature which did not allow for a standardised approach to all cases and for the description and the notes written by the clinicians. Moreover, the lack of standardisation led to incomplete medical records and inability to find information regarding all the parameters evaluated in every patient.

Adding further cases to the model, would increase its accuracy, and would allow the identification of other potential differentiating factors between groups. In cases with electrolyte abnormalities, it is recommended to do an ACTHst, instead of just checking RC. A study comparing only patients without electrolyte abnormalities, with and without HA, would possibly provide more conclusive data. Nonetheless, results of this study allow for its use in daily clinical practice, helping the decision-making process of the clinicians.

In conclusion, this study observed that multiple variables are associated with an increased likelihood of HA diagnosis, particularly lethargy, anorexia, and low sodium and albumin concentrations. These findings provide new insights and assist clinicians in better assessing the urgency of performing an ACTHst, especially in cases where routine RC is used, such as in the medical investigation of chronic gastrointestinal signs, where the prevalence of HA is low.

## Data Availability

The raw data supporting the conclusions of this article will be made available by the authors, without undue reservation.
