# Supplementary material for: Can we predict hypoadrenocorticism in dogs with resting hypocortisolemia? A predictive model based on clinical, haematological, and biochemical variables
Source: Front Vet Sci. 2024 Dec 23;11:1523170. doi: 10.3389/fvets.2024.1523170 (PMC11700806; doi:10.3389/fvets.2024.1523170)
Supplement: Supplementary file 1 [file Table_1.DOCX]

Supplementary file

Table A1: Haematology and serum biochemistry by disease status (results are expressed as median with the respective interquartile range; *Mann-Whitney U Test to compare laboratory variables regarding disease group)

| Variable | **nHA group**  **(n=61)** | **HA group**  **(n=29)** | ***P**** |
| --- | --- | --- | --- |
| Albumin (g/dl) | 3.2 (0.37) | 2.88 (0.5) | <0.001 |
| ALP  (U/L) | 46.0 (59.0) | 26.9 (70.0) | 0.26 |
| ALT  (U/L) | 46.5 (33.2) | 67.0 (64.3) | 0.01 |
| BUN  (mg/dl) | 33.8 (15.7) | 74.0 (78.0) | <0.001 |
| Creatinine  (mg/dl) | 1.08 (0.41) | 1.50 (0.79) | <0.001 |
| Eosinophils  (absolute value) | 340.0 (464.0) | 330.0 (445.0) | 0.99 |
| Erythrocytes  (M/µL) | 7.03 (1.2) | 7.37 (1.55) | 0.17 |
| Glucose  (mg/dl) | 96.0 (18.0) | 93.10 (39.8) | 0.44 |
| GGT  (U/L) | 3.70 (2.86) | 4.70 (3.11) | 0.41 |
| Hematocrit  (%) | 47.6 (9.6) | 48.5 (12.7) | 0.93 |
| Leucocytes  (x10^3^/ µL) | 10.8 (4.58) | 11.74 (7.18) | 0.09 |
| Lymphocytes  (absolute value) | 2048.0 (1582.0) | 2840.0 (1450.0) | 0.05 |
| NaK Ratio | 33.0 (4.4) | 27.50 (12.6) | <0.001 |
| Neutrophils  (absolute value) | 7018.0 (3297.0) | 6700.0 (6948.0) | 0.25 |
| Potassium  (mmol/L) | 4.50 (0.7) | 5.30 (2.0) | 0.004 |
| Sodium  (mmol/L) | 148.0 (6.0) | 136.0 (21.0) | <0.001 |
